# Zinc Oxide Nanoparticles Induced Oxidative DNA Damage, Inflammation and Apoptosis in Rat’s Brain after Oral Exposure

**DOI:** 10.3390/toxics6020029

**Published:** 2018-05-26

**Authors:** Hala Attia, Howaida Nounou, Manal Shalaby

**Affiliations:** 1 Department of Pharmacology and Toxicology, College of Pharmacy, King Saud University, Riyadh 11495, Saudi Arabia; hsalem@ksu.edu.sa; 2 Department of Biochemistry, Faculty of Pharmacy, Mansoura University, Mansoura 35516, Egypt; 3 Department of Medical Biochemistry, Faculty of Medicine, Alexandria University, Alexandria 21111, Egypt; 4 Department of Medical Biotechnology, Institute of Genetic Engineering City of Scientific Research and biotechnological applications, Borg El Arab, Alexandria 21111, Egypt; manalshalaby2870@yahoo.com

**Keywords:** ZnONPs, brain, DNA fragmentation, oxidative stress, heat shock protein-70, interleukin-1β, caspase-3, Fas

## Abstract

Growing evidences demonstrated that zinc oxide nanoparticles (ZnONPs) could reach the brain after oral ingestion; however, the “neurotoxicity of” ZnONPs after oral exposure has not been fully investigated. This study aimed to explore the “neurotoxicity of” ZnONPs (<100 nm) after oral exposure to two doses; 40 and 100 mg/kg for 24 h and 7 days. The exposure to 40 and 100 mg/kg of ZnONPs for 24 h did not elicit “neurotoxicity” compared to normal control. However, the daily exposure to both doses for 7 days caused oxidative stress in brain tissue as detected by the elevation of the levels of malondialdehyde, the main product of lipid peroxidation and nitrite as an index of nitric oxide with concomitant decline in the concentrations of antioxidants. In addition, both doses resulted in DNA fragmentation which was confirmed by increased percentage of tailed DNA, DNA tail intensity and length and tail moment particularly with the dose 100 mg/kg. Moreover, both doses led to the elevation of the inflammatory cytokines along with increased apoptotic markers including caspase-3 and Fas. Heat shock protein-70 levels were also elevated possibly as a compensatory mechanism to counteract the ZnONPs-induced oxidative stress and apoptosis. The present results indicate the “neurotoxicity of” ZnONPs after recurrent oral exposure via oxidative stress, genotoxicity, inflammatory response and apoptosis.

## 1. Introduction

Metallic (metal or metal oxide) nanoparticles (NPs) have unique physicochemical features including surface charge, size, and shape that enable them to integrate with biological systems with high specificity [1]. However, the expansion of NPs usage in industry, medicine, energy, biotechnology, cosmetics and other fields leads to an increased “toxicity” for human [1]. The consumption of nanostructured ingredients in numerous human fields has directed investigators to examine their toxic effects upon getting to the living organisms through different routes such as ingestion, respiration or skin penetration [2].

Growing evidences revealed that once NPs enter the body through different routes including the oral administration, they could be absorbed and distributed by systemic circulation to various tissues [3,4,5] including the brain. Direct disruption of neuronal cell membranes by NPs would allow their entry into the brain, subsequently they could elicit toxic effects [6,7].

Among various metal oxides, zinc oxide nanoparticles (ZnONPs) are the most widely used NPs for their unique physical and chemical properties and easy synthesis [8]. ZnONPs are used in a variety of products such as dyes, toothpaste, cosmotics, sunscreens, textiles, wall paints, and other building materials [9,10]. In addition, ZnONPs are used in the food industry as additives owing to their antimicrobial potential [11], in agriculture as antifungal [12] and medically as anticancer [13]. Therefore the oral exposure to ZnONPs is increased alarmingly. 

Several in vivo studies demonstrated that ZnONPs could reach various organs after systemic distribution and have been shown to elicit cytotoxicity in different animal organs including liver, heart, kidney, lung, spleen, and pancreas [14,15,16,17,18,19,20]. In addition, the toxic effects have also been documented in many cultured cells such as human blood cells [21]; primary human keratinocytes [22] vascular endothelial cells [23], macrophages [24] and lung epithelial cells [25]. According to these earlier studies, the “toxicity of” the ZnONP is attributed mainly to the production of reactive oxygen species (ROS), inflammation, genotoxicity and cell death.

Several features could explain the mechanisms of ZnO NPs toxicity on living organisms including; the physical injury due to direct contact, the zinc ions that have been dissolved and the mechanism mediated by reactive oxygen species (ROS) [26].

Although the adverse effects of ZnONPs have extensively studied on various organs and cell lines, the “toxicity” on brain tissue is not fully investigated. Recently, in vitro studies revealed that ZnONPs induced oxidative stress, cell death and energy depletion in microglia cell line [27,28]. In addition, in vitro studies, demonstrated that ZnONPs induced the apoptosis of neural stem cell [29] and disturbed the ion channel current in primary hippocampal neurons [30]. Earlier, ZnONPs have shown “toxicity” in mice brain tumor cell lines when compared with similar sized particles of Al_2_O_3_, TiO_2_, Fe_3_O_4_, and CrO_3_ [31]. However, the in vitro studies have restricted ability to imitate in vivo systems, which contain many complicated NPs–biosystems interactions that influence the pharmacokinetics of NPs such as biodistribution and metabolism, in addition to the tissue response. Therefore, it is essential to study the neurotoxic effects of ZnONPs in vivo to emphasize the in vitro studies.

It has been detected that ZnONPs can reach the brain after oral ingestion either by breaking the blood brain barrier (BBB) or by neural transportation [32,33]. Shim et al. [34] demonstrated that ZnONPs interact with plasma and brain leading to toxic effects in the blood and brain. Among different proteins detected on the surface of ZnONPs, apolipoprotein E was present, which is known to mediate the passing of NPs across the BBB and to be entangled in neurological diseases. 

Although the oral exposure to ZnONPs is increased alarmingly, the studies investigating its “neurotoxic” effects after oral ingestion are scanty. One recent study was done by Xiaoli et al. [35] on rat offspring, demonstrated imbalanced antioxidant status and apoptotic death in brain cells. In the present study the neurotoxic effects of ZnONPs were evaluated in mature rats’ brain tissue after oral exposure by gavage. The studied underlying mechanisms included oxidative stress, genotoxicity, inflammation and apoptosis. 

## 2. Materials and Methods

### 2.1. Chemicals and Kits 

ZnO-NPs (<100 nm, surface area 15–25 m^2^/g, purity >99%, Cat. No. 544906), thiobarbituric acid (TBA), sulfanilamide, N-(1-naphthyl) ethylenediamine dihydrochloride and Ellman’s reagent (dithiobis-2-nitrobenzoic acid, DTNB) were supplied by Sigma-Aldrich chemical Co. (St Louis, MO, USA). Kit for the assay of superoxide dismutase (SOD) was obtained from Cayman Chemical Co. (Ann Arbor, MI, USA). Tumor necrosis factor-α (TNF-α) and interleukin-1β (IL-1β) were measured using rat ELISA kits from R&D Co. (Quantikine, R&D systems, Minneapolis, MN, USA). Fas and Caspase-3 rat ELISA kits were purchased from Cloud-Clone Corp Co. (Houston, TX, USA). Heat shock protein-70 (HSP-70) levels were measured using rat ELISA kit obtained from MyBioSource, Inc. (San Diego, CA, USA). All other chemicals were of high analytical grade. 

### 2.2. Particle Characterization

ZnONPs suspension was prepared as described in our previous work [18]. The morphology and particle size were determined with transmission electron microscopy (TEM). The hydrodynamic size, size distribution and the surface zeta potential were estimated using dynamic light scattering (DLS). 

### 2.3. Study Design

ZnONPs was suspended in normal saline and administered to rats by oral gavage tube according to body weight. Sixty Wistar albino adult male rats, aged between 10–11 weeks, as the brain is well developed at this age [36], weighing 180–230 g were kept in standard conditions (a 12-h light/dark cycle, temperature of 22 °C ± 2 °C and relative humidity 50% ± 20%). They were served with standard food pellets and tap water *ad libitum*. The experimental procedures were conducted according to the National Institute of Health Guide for Animal Care and approved by the local Ethics Committees at both Alexandria University and King Saud University.

Table 1 shows rats administered ZnONPs classified into four groups (*n* = 10) as follows: Groups I and II treated with 40 and 100 mg/kg ZnONPs, respectively for 24 h, while groups III and VI received 40 and 100 mg/kg ZnONPs, respectively daily for 7 days. The doses were selected based on our previous work on lung tissue [18] in which both doses induced oxidative stress, inflammation and damage of DNA after 24 h and more pronounced effect after 7 days. Two normal control groups (A and B) were used. Control A received normal saline for 24 h by oral gavage tube, and control B was given normal saline daily for 7 days by oral gavage tube.

### 2.4. Processing of Brain Tissue for Biochemical Studies

At the end of treatment (24 h and 7 days); rats were anesthetized with isoflurane and sacrificed by decapitation. Brains were isolated and rinsed with ice-cold phosphate buffer saline (PBS), pH 7.4 for several times to remove the excess blood and any clots. Brain was divided into parts, then blotted individually on ash –free filter paper and weighed. A part of each brain was homogenized with PBS, pH 7.4 (1:5 *w*/*v*) in an Ultra-Turrax homogenizer. The homogenate was then centrifuged for 10 min at 3000 rpm, 4 °C and supernatants were allocated into aliquots and kept at −80 °C for the subsequent assessment of oxidative stress and inflammatory markers.

For assessment of SOD activity, another part of brain tissue was homogenized in 10 mL cold 20 mM 4-2-hydroxyethyl-1-piperazineethanesulfonic acid (HEPES) buffer, pH 7.2, containing 1 mM ethylene glycol tetraacetic acid (EGTA), 210 mM mannitol and 70 mM sucrose per g tissue as specified by the instructions of the assay kit used. For assessment of HSP-70 and apoptotic markers; 500 mg of each brain tissues was minced to small pieces and homogenized in 500 µL of PBS (pH 7.2) for HSP-70 and Fas assay, while fresh lysis buffer (50 mM HEPES, pH 7.5, 0.1% 3-[(3-**ch**olamidopropyl)dimethyl**a**mmonio]-1-**p**ropane**s**ulfonate (CHAPS), 2 mM dithiothreitol, 0.1% Nonidet P-40, 1 mM EDTA, 1 mM phenylmethylsulfonyl fluoride, 2 μg/mL leupeptin, and 2 μg/mL pepstatin A) *w*:*v* = 1:20 was used for caspase-3 assay, using a glass homogenizer on ice. After that, the homogenates were centrifuged for 15 min at 5000 rpm, 4 °C. The supernatant was collected, divided into aliquots and kept at −80 °C for the subsequent assessment of HSP-70, Fas and caspase-3.

### 2.5. Assessment of Oxidative Stress Markers (Lipid Peroxidation and Antioxidants) in Brain Tissue

Lipid peroxidation leads to the formation of malondialdehyde (MDA) which is a main product in the sequence of polyunsaturated fatty acids oxidation. The lipid peroxides produced were measured by the increase in thiobarbituric acid reactive substances (TBARS) levels using TBA reagent as described previously [37]. The absorbance of the developed pink-colored product was measured at 535 nm against a reagent blank. 

Reduced glutathione (GSH, a non-enzymatic antioxidant) was estimated using Ellman’s reagent as specified by the technique described by Moron et al. [38] with some modification. The absorbance of the generated yellow color was read at 412 nm. 

Catalase (CAT, an enzymatic antioxidant) activity was assessed as the rate of decomposition of hydrogen peroxide (H_2_O_2_) [39]. Briefly, 500 µL 1% H_2_O_2_ in PBS was added to 5 µL of homogenate, then the mixture was incubated for 10 min at 28 °C and the rate of decomposition of H_2_O_2_ was measured at 240 nm. 

The assay of SOD activity using Cayman’s Superoxide Dismutase Assay Kit was based on the use of tetrazolium salt for detection of the superoxide radicals produced by xanthine oxidase. The quantity of the enzyme required to exhibit 50% dismutation of the superoxide radicals represents one unit of SOD activity. This SOD assay was established on basis of the scheme of Kakkar et al. [40]. Briefly, 200 µL of the diluted radical detector (tetrazolium salt) and 10 µL of either SOD standards or the brain homogenate samples were added per well. Then the reaction was initiated by adding 10 µL of diluted xanthine oxidase to every well. The plate was shaked cautiously for few seconds to mix, and sealed with plate cover. The plate was incubated on the shaker at room temperature for 30 min. The absorbance was read in the plate reader at 450 nm. The results were stated as unit/g of tissue.

### 2.6. Measurement of Nitrite (an Index of Nitric Oxide) 

The biologically produced nitric oxide (NO) is rapidly oxidized to nitrite and nitrate, therefore, nitrite levels can reflect NO production. Nitrite was assessed colorimetrically using Griess reagent (a mixture of equal volumes of 1% sulfanilamide in orthophosphoric acid (2.5%) and 0.1% N-(1-naphthyl) ethylenediamine in distilled water) [41]. The reaction mixture was 100 µL of brain homogenate and 100 µL Griess reagent, incubated for 10 min at room temperature. The absorbance of the developed orange color was read at 540 nm. 

### 2.7. Assay of HSP-70 in Brain Tissues

HSP-70 ELISA kit applies the competitive enzyme immunoassay technique utilizing a monoclonal anti-HSP-70 antibody and HSP-70- horseradish peroxidase (HRP) conjugate. After incubation of the assay samples and buffer altogether with HSP-70-HRP conjugate in pre-coated plate, for one hour, the wells were decanted and washed five times. Then, the wells were incubated with tetramethylbenzidine (TMB), a substrate for HRP enzyme. A blue colored complex was formed as a product of the enzyme-substrate reaction. Lastly, the solution was turned yellow after the addition of a stop solution. The intensity of color was estimated spectrophotometrically in a microplate reader at 450 nm. A standard curve was plotted relating the color intensity to the concentration of standards. The concentration of HSP-70 in every sample was interpolated out of the standard curve [42].

### 2.8. Assessment of Inflammatory Markers (TNF-α & IL-1β) in Brain Tissues 

#### 2.8.1. Measurement of IL-1β 

This assay employs the quantitative sandwich enzyme immunoassay technique utilizing microplate pre-coated with polyclonal antibody specific for rat IL-1β. After pipetting each of the standards and brain homogenate of samples as well as controls into the wells, the immobilized antibody bound any existing rat IL-1β. Then, any unbound substances were washed away and an enzyme-linked polyclonal antibody specific for rat IL-1β was added to the wells. After washing, to remove any unbound antibody enzyme reagent, a substrate solution (TMB) was then added to the wells. The enzyme reaction yielded a blue color that changed to yellow after the addition of the stop solution. The intensity of the color was measured. It was directly proportional to the quantity of rat IL-1β bound in the first step. The sample concentrations were then read out of the standard curve [43]. 

#### 2.8.2. Measurement of Tumor Necrosis Factor-α (TNF-α) 

Similar assay procedure for estimation of IL-1β, described previously, was applied for the estimation of TNF-α utilizing microplate pre-coated with a specific monoclonal antibody for rat TNF-α [44]. 

### 2.9. Assay of Apoptotic Markers (Fas & Caspase-3) in Brain Tissues 

These assays employ the quantitative sandwich enzyme immunoassay technique utilizing microplates pre-coated with antibodies specific to either Fas or casapse-3. Each of the standards and the brain samples was added to the appropriate microplate wells with a biotin-conjugated antibody specific to either Fas or casapse-3. Then, avidin conjugated to HRP was added to every microplate well and incubated. After TMB substrate solution was added, only those wells that contain Fas or casapse-3, biotin-conjugated antibody and enzyme-conjugated avidin exhibited change in color. Sulphuric acid solution was added to terminate the enzyme-substrate reaction and the color transformation was assessed spectrophotometrically at a wavelength of 450 nm. The concentrations of either Fas or casapse-3 in the samples were then determined by comparing the O.D. of the samples to the standard curve [45,46].

### 2.10. Assay of DNA Damage Markers

#### Measurement of DNA Fragmentation

DNA fragmentation was quantified using diphenylamine (DPA) reagent according to the technique of Burton [47] and modified by Suenobu et al. [48]. Brain homogenate was lysed with 0.4 mL lysis buffer (10 mmol/L Tris, 1 mmol/L EDTA, and 0.1% NP-40, pH 7.5) and centrifuged at 13,000× *g* for 10 min to isolate the intact chromatin from the fragmented one. The fragmented DNA, in the supernatant, was transferred to a distinct microfuge tube, and both supernatant and pellet were precipitated overnight at 4 °C in 12.5% trichloroacetic acid (TCA). The precipitates were sedimented at 13,000× *g* for 4 min. The DNA precipitates were hydrolyzed by heating to 90 °C for 10 min in 5% TCA. For quantification of fragmented DNA, in brief, 0.16 mL of DPA reagent (0.15 g DPA, 0.15 mL H_2_SO_4_, and 0.05 mL acetaldehyde per 10 mL glacial acetic acid) was added to each tube and the absorbance of the overnight developed color was measured at 570 nm. “Percent fragmentation” indicates the proportion of DNA in the supernatant (“fragmented”) to the total DNA recovered in both supernatant and pellet (“fragmented plus intact”).

### 2.11. Comet DNA Assay

The level of DNA damage was determined as described by Singh et al. [49]. First, to prepare the brain homogenate, a small part of brain tissue was crushed and transferred to 1 mL ice-cold PBS and homogenized at 500 rpm in ice. For the assay, 600 μL of 0.8% low-melting agarose was added to 100 μL of brain homogenate and then 100 μL of this mixture was spread on slides pre-coated with 300 μL of normal melting agarose and immersed in lysis buffer (0.045 M trisborate-EDTA (TBE), pH 8.4) for 15 min. Slides were then placed for 20 min in a horizontal electrophoresis unit at alkaline pH (1 mM Na_2_EDTA and 300 mM NaOH, pH 13) to allow the unwinding of DNA. The electrophoresis conditions were 25 V, 300 mA, and distance between electrodes 30 cm for 20 min at room temperature in the formerly mentioned alkaline solution (pH 13). The slides were stained using ethidium bromide (20 μg/mL) at 4 °C, covered and kept in sealed boxes at 4 °C up to image analysis. To avoid extra DNA damage by UV, all steps were achieved under dimmed light. For each sample, images of 100 arbitrarily chosen cells were examined. DNA fragment migration patterns were evaluated with a fluorescence microscope (magnification 250×). DNA damage was evaluated as tail length (TL = distance of DNA migration from the center of the body of the nuclear core), tail intensity (TI = % of DNA that migrated from the nuclear core to the tail) and tail moment. The tail moment (TMOM) was determined according to the formula: TMOM = DNA in tail as a % of total DNA × tail length (TL). 

### 2.12. Statistical Analysis

Data are expressed as mean ± SEM. Statistical comparisons between groups were accomplished using one way analysis of variance (ANOVA) followed by Tukey Krammer test as post multiple test. Statistical analysis was done using Graph Pad Prism 4 software Inc., San Diego, CA, USA. Results were considered significant at *p* < 0.05.

## 3. Results

### 3.1. Particle Characterization

As described in our previous work [18], the TEM images showed that ZnONPs had a spheroid shape and no aggregation was detected. The mean particle size was 30 ± 1.12 nm; the mean hydrodynamic diameter was 272 nm ± 8.75 and the surface zeta potential was –41.2 ± 0.65 mV. 

### 3.2. Biochemical Measurements

No differences were observed upon comparing the results of the control group A (for 24 h) with the control group B (for 7 days), so for simplicity the two groups were combined in one control group.

### 3.3. Effect of ZnONPs on the Oxidative Stress Markers in Brain Tissue 

**Figure 1:**
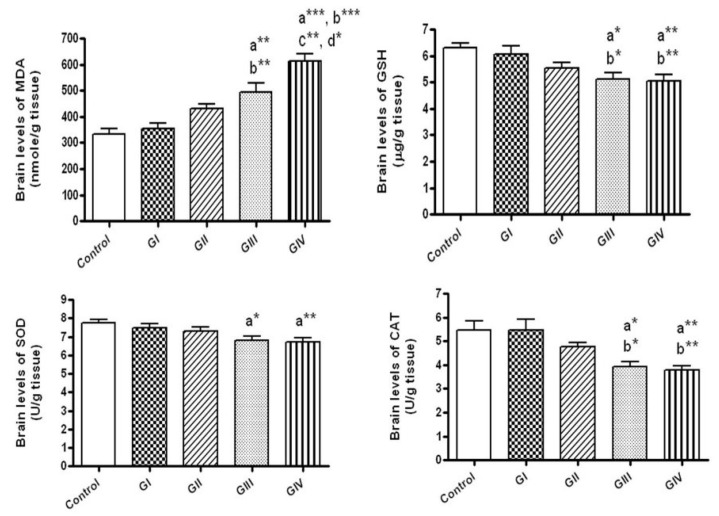
The brain levels of oxidative stress markers in normal control and different ZnONPs-treated groups. Data are expressed as mean ± SEM. a: significantly different from normal control; b: significantly different from G I; c: Significantly different from G II; d: Significantly different from G III. * *p* < 0.05; ** *p* < 0.01; *** *p* < 0.001. MDA: Malondialdehyde (the index of lipid peroxidation), GSH: Reduced glutathione (a non-enzymatic antioxidant), SOD: superoxide dismutase; CAT: Catalase (enzymatic antioxidants). G I & G II: rats treated with 40 and 100 mg/kg for 24 h, respectively. G III & G IV are rats treated with 40 and 100 mg/kg/day for 7 days, respectively.

After 24 h, no alterations were observed in the brain levels of MDA, GSH, SOD, CAT and nitrite upon treating either by 40 or 100 mg/kg of ZnONPs in comparison to control group. In contrary, the treatment with these doses daily for 7 days resulted in significant elevation of MDA (*p* < 0.01, *p* < 0.001, respectively) together with significant reductions of the antioxidants; GSH, CAT and SOD in brain tissues when compared to control group (*p* < 0.05, *p* < 0.01, for the doses 40 and 100 mg/kg respectively) (Figure 1). 

As shown in Figure 1, daily administration of 40 and 100 mg/kg ZnONPs for 7 days (Groups III and group IV, respectively) revealed significant rise in the brain levels of MDA (*p* < 0.01, *p* < 0.001, respectively) with significant decline in GSH (*p* < 0.05, *p* < 0.01, respectively) and CAT (*p* < 0.05, *p* < 0.01, respectively) in comparison to group I (40 mg/kg for 24 h). In addition, MDA levels revealed significant rise in rats given 100 mg/kg daily for 7 days (group IV) in comparison to those given 100 mg/kg for 24 h (group II, *p* < 0.01) and those administered 40 mg/kg daily for 7 days (group III, *p* < 0.05).

**Figure 2:**
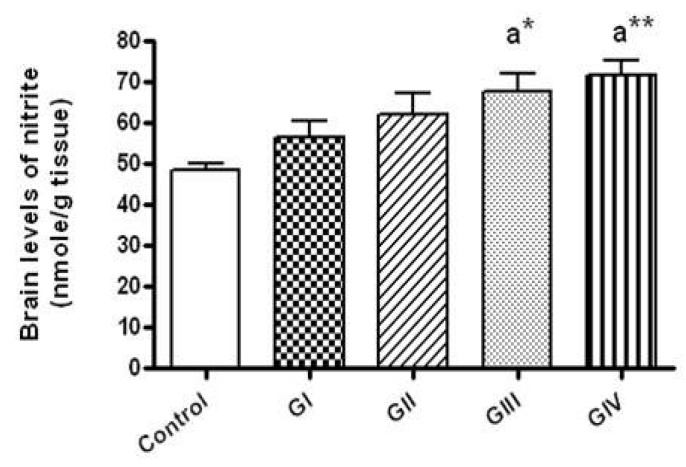
The brain levels of nitrite (an index of nitric oxide production) in normal control and different ZnONPs-treated groups. Data are expressed as mean ± SEM. a: significantly different from normal control; * *p* < 0.05; ** *p* < 0.01. G I & G II: rats treated with 40 and 100 mg/kg for 24 h, respectively. G III & G IV are rats treated with 40 and 100 mg/kg/day for 7 days, respectively.

Regarding the brain concentrations of nitrite, rats received 40 and 100 mg/kg daily for 7 days showed significant increment of brain nitrite levels in comparison to the control (*p* < 0.05, *p* < 0.01, respectively). No significant differences in nitrite levels were observed between different ZnONPs—treated groups (Figure 2).

### 3.4. Effect of ZnONPs on the Inflammatory Markers in Brain Tissue 

**Figure 3:**
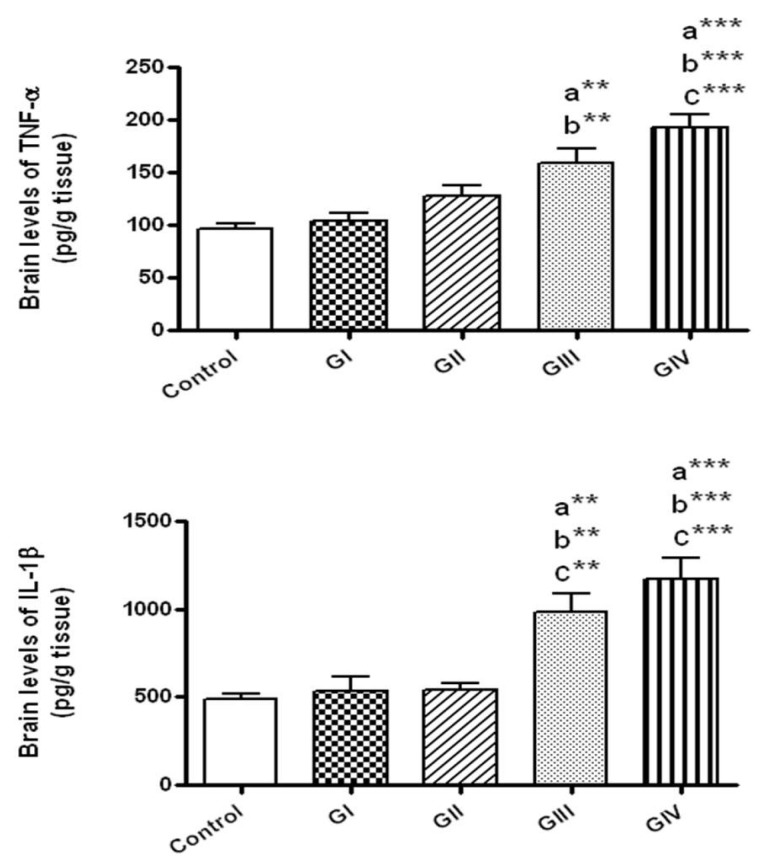
The brain levels of the inflammatory markers in normal control and different ZnONPs-treated groups. Data are expressed as mean ± SEM. a: Significantly different from normal control; b: Significantly different from G I; c: significantly different from G II. ** *p* < 0.01; *** *p* < 0.001. TNF-α: tumor necrosis factor-α; IL-1β: interleukin-1β. G I & G II: Rats treated with 40 and 100 mg/kg for 24 h, respectively. G III & G IV are rats treated with 40 and 100 mg/kg/day for 7 days, respectively.

The brain levels of TNF-α and IL-1β were markedly elevated only in the groups treated with ZnONPs daily for 7 days either at the dose 40 mg/kg/day (Group III) or 100 mg/kg/day (group IV) in comparison to control group (*p* < 0.01 and *p* < 0.001, respectively). In addition, TNF-α and IL-1β concentrations were significantly raised in the group exposed to 100 mg/kg daily for 7 days in comparison to those exposed to 40 and 100 mg/kg for 24 h (groups I and II, respectively, *p* < 0.001). Rats given 40 mg/kg daily for 7 days revealed significant rise in TNF-α levels in comparison to group I (*p* < 0.01) and in IL-1β levels compared to groups I and II (*p* < 0.01) (Figure 3).

### 3.5. Effect of ZnONPs on HSP-70 in Brain Tissue 

**Figure 4:**
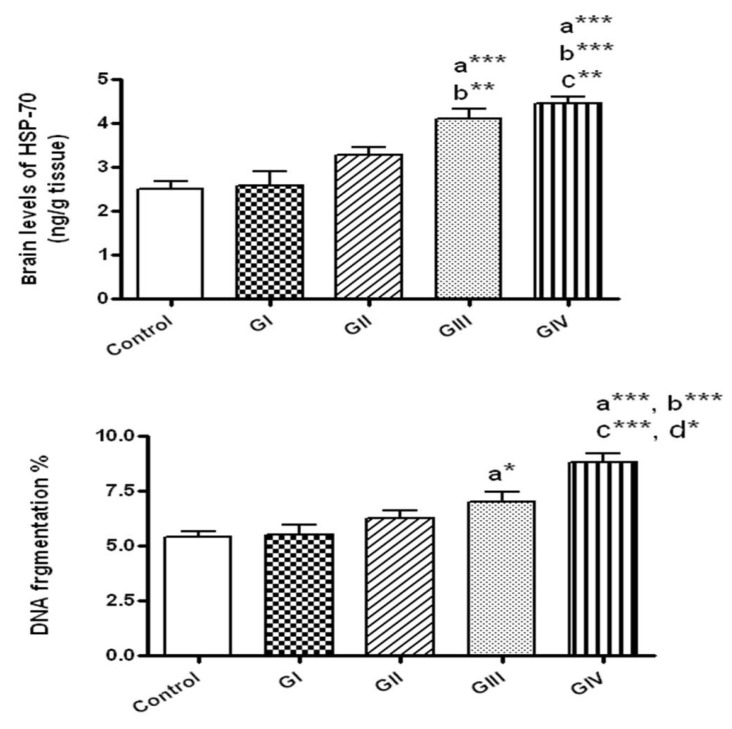
The brain levels of heat shock protein-70 (HSP-70) and DNA fragmentation in normal control and different ZnONPs-treated groups. Data are expressed as mean ± SEM. a: Significantly different from normal control; b: significantly different from G I; c: significantly different from G II; d: Significantly different from G III. * *p* < 0.05; ** *p* < 0.01; ***p < 0.001. G I & G II: rats treated with 40 and 100 mg/kg for 24 h, respectively. G III & G IV are rats treated with 40 and 100 mg/kg/day for 7 days, respectively.

After 24 h, the concentrations of HSP-70 did not change by the exposure to either 40 or 100 mg/kg (groups I and II, respectively) in comparison to control. However, HSP-70 concentrations were markedly elevated in rats received 40 mg/kg daily for 7 days (group III) in comparison to control (*p* < 0.001) and to the group I that received 40 mg/kg for 24 h (*p* < 0.01). Rats given 100 mg/kg/day for 7 days showed significant elevation of HSP-70 compared to normal control (*p* < 0.001) and to the groups received 40 and 100 mg/kg for 24 h (*p* < 0.001 and *p* < 0.01, respectively). No significant alterations were detected in HSP-70 levels in rats received 100 mg/kg daily for 7 days in comparison to those given 40 mg/kg daily for 7 days (Figure 4). 

### 3.6. Effect of ZnONPs on DNA Fragmentation in Brain Tissue 

As presented in Figure 4, DNA fragmentation % was significantly augmented only in rats received ZnONPs for 7 days at the doses 40 and 100 mg/kg/day in comparison to control (*p* < 0.05, *p* < 0.001, respectively). In addition, DNA fragmentation was significantly augmented in rats treated for 7 days with 100 mg/kg in comparison to those exposed to 40 mg/kg daily for 7 days (*p* < 0.05) and to 40 and 100 mg/kg for 24 h (*p* < 0.001). 

### 3.7. Effect of ZnONPs on DNA Comet Assay Indices in Brain Tissue 

**Figure 5:**
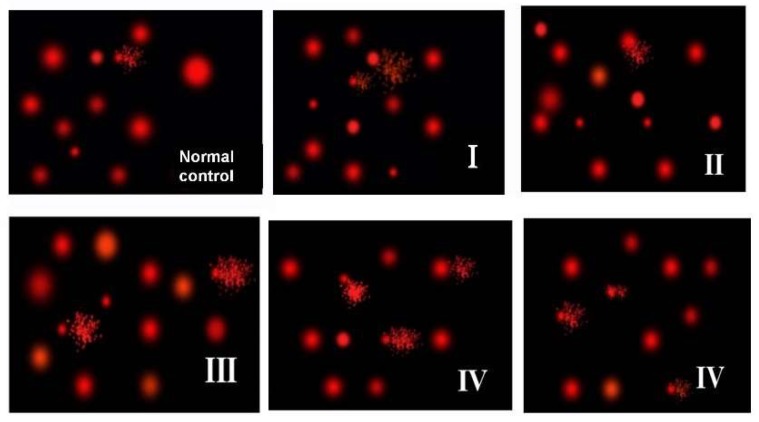
Comet DNA assay in brain tissues. G I & G II: rats treated with 40 and 100 mg/kg for 24 h, respectively. G III & G IV are rats treated with 40 and 100 mg/kg/day for 7 days, respectively.

There are no changes in all comet assay indices (tailed DNA %, tail length, tail intensity and tail moment) in the brain of rats exposed to 40 and 100 mg/kg for 24 h in comparison to control. Brain of rats received 40 mg/kg daily for 7 days revealed significant elevation in tail length and tail moment in comparison to control (*p* < 0.05), while, no significant alterations were detected in tailed DNA % or tail intensity. Brain of rats exposed to 100 mg/kg daily for 7 days showed remarkable increase in tailed DNA % (*p* < 0.01), tail length (*p* < 0.001), tail intensity (*p* < 0.01) and tail moment (*p* < 0.001) in comparison to control. This group also showed significant rise in tail length, intensity and moment compared to rats given 40 and 100 mg/kg/24 h and those given 40 mg/kg daily for 7 days. A significant increase in the tailed DNA % was observed in the brain of rats given 100 mg/kg daily for 7 days in comparison to those given 40 mg/kg for 24 h only (*p* < 0.05) (Figure 5 & Table 2). 

### 3.8. Effect of ZnONPs on Apoptotic Markers in Brain Tissue 

**Figure 6:**
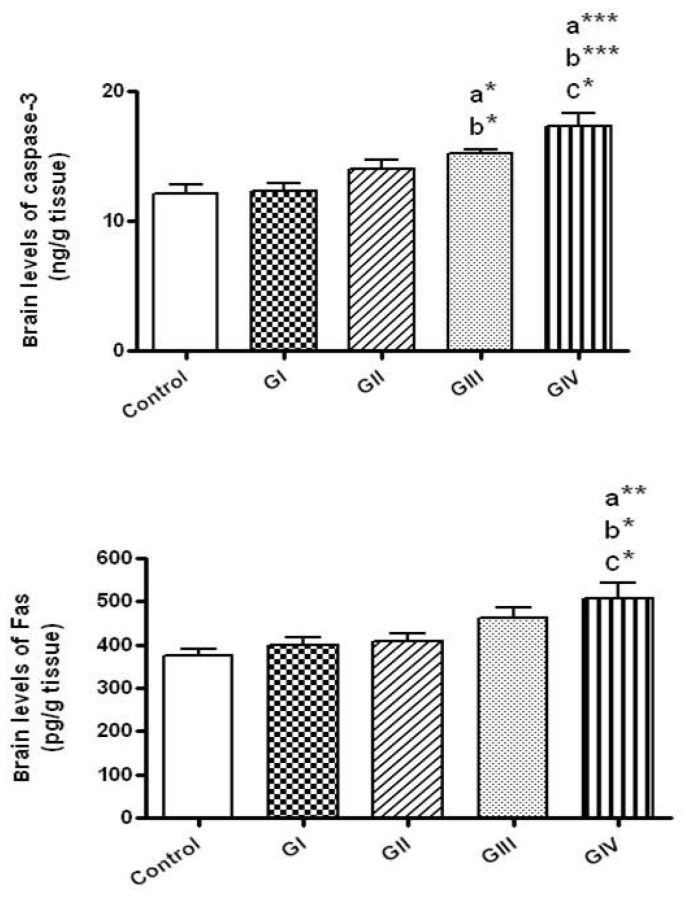
The brain levels of apoptotic markers (caspase-3 and Fas) in normal control and different ZnONPs-treated groups. Data are expressed as mean ± SEM. a: significantly different from normal control; b: significantly different from G I; c: significantly different from G II. * *p* < 0.05; ** *p* < 0.01; *** *p* < 0.001. G I & G II: rats treated with 40 and 100 mg/kg for 24 h, respectively. G III & G IV are rats treated with 40 and 100 mg/kg/day for 7 days, respectively.

No significant alteration in the brain levels of casapse-3 (the executioner apoptotic protein) was shown in rats received ZnONPs for 24 h either by the dose 40 mg/kg (Group I) or at 100 mg/kg (Group II). However, significant elevations of casapse-3 levels were detected in rats treated for 7 days by either 40 mg/kg/day (Group III, *p* < 0.05) or by 100 mg/kg/day (Group IV, *p* < 0.001) in comparison to control. Rats received ZnONPs at the dose 40 mg/kg daily for 7 days showed higher caspae-3 levels in comparison to those given 40 mg/kg for 24 h (*p* < 0.05). The brain concentrations of caspase-3 were markedly higher in rats received 100 mg/kg daily for 7 days in comparison to those received 100 mg/kg for 24 h (Group II, *p* < 0.05) and to those given 40 mg/kg for 24 h (Group I, *p* < 0.001) (Figure 6).

Concerning the extrinsic apoptotic protein, Fas, their brain levels did not change in rat groups treated with ZnONPs for 24 h either at 40 or 100 mg/kg and in the group treated with 40 mg/kg daily for 7 days in comparison to the control group. However, brain Fas levels were significantly elevated by the treatment for 7 days with 100 mg/kg/day in comparison to control (*p* < 0.01) and to rats exposed to 40 and 100 mg/kg for 24 h (*p* < 0.05) (Figure 6).

## 4. Discussion

Despite the many advantages of NPs, they may cause hazardous effects because of their special properties including small size and high surface area [1]. It has been shown that NPs may cause injury to the BBB leading to increased permeability and BBB disruption and penetration of NPs, causing neurotoxicity [6,7,33]. 

The extensive use of ZnONPs in food industry, agriculture and medicine increases their contact with various organs and the subsequent cytotoxicity after oral exposure. Among these organs, ZnONPs could enter the brain after oral ingestion by either the disruption of BBB or via neural transportation [32,33]. However, studies investigating the “neurotoxicity of” ZnONPs due to oral ingestion are scanty. Recently, the prenatal oral exposure to ZnONPs resulted in imbalanced antioxidant status and apoptotic death in the offsprings’ brain cells [35]. In addition, Ansar et al. [50] reported that the oral ingestion of high dose (600 mg/kg) of ZnONPs for 7 days resulted in oxidative stress and inflammation in rat’s brain tissue. In the current work we used mature rats to study the “neurotoxicity of” oral exposure to two low doses; 40 and 100 mg/ kg; of ZnONPs (100 nm) for 24 h and 7 days. Previously, we demonstrated that these doses elicited severe pulmonary “toxicity” upon oral administration [18] for 24 h and 7 days. The current data revealed a “neurotoxicity of” these doses only after 7 days while, no significant hazardous effects were detected after 24 h, suggesting the “neurotoxicity” by the accumulation of ZnONPs in the brain or that ZnONPs do not distributed into the brain after 24 h. These data may confirm the previous work revealing that, at 24 h, Zn content was mainly distributed in the kidney, liver, and lung, while at ≥7 days, it was mainly distributed in the kidney, liver, lung, and brain. Furthermore, as time increases, Zn contents decreases in the kidney and liver, but increases in the brain [51]. 

The enzymatic antioxidants; SOD and CAT work together to ameliorate the oxidative stress. SOD catalyzes the dismutation of superoxide radical (O_2_^•−^) into H_2_O_2_, which in turn is decomposed to water and oxygen by CAT [52]. GSH is a non-enzymatic antioxidant which plays an essential role in scavenging the free radicals via its –SH group [53]. Our data showed that oral exposure to 40 and 100 mg/kg of ZnONPs daily for 7 days led to significant depletion of the concentrations of the antioxidants GSH, SOD and CAT indicating that ZnONPs deteriorate the antioxidant system in the brain tissue with the subsequent oxidative and nitrosative stress. This is consistent with previous report showing that exposure of fish; Oreochromis niloticus and Tilapia zillii to 2 mg/L silver nanoparticles (Ag-NPs) in water did not produce a significant change in GSH and total (tGSH) levels in brain tissue, while exposure to 4 mg/L produced a significant decrease in GSH and tGSH contents. SOD and CAT activity and gene expression also deteriorated in the brain of fish exposed to the highest Ag-NPs concentrations [54].

The increase in TBRRS and nitrite levels in the brain reflects this stress. The depletion in GSH, SOD and CAT in ZnONPs-treated rats could be related to their consuming during the elimination of the generated ROS. The enhanced production of ROS cause lipid peroxidation by converting polyunsaturated fatty acids in the cell membrane to toxic lipid peroxides destroying the membrane and causing cell injury [53]. The increase in TBARS formation is an index of lipid peroxidation. This was supported by our findings, which revealed rise of TBARS at the doses 40 and 100 mg/kg/day for 7 days. Moreover, nitric oxide (NO) can be cytotoxic by interaction with oxygen forming nitrosative species such as peroxynitrite which leads to damage to macromolecules like DNA and lipids by oxidation, nitrosation or nitration [55]. After 7 days, the high concentrations of nitrite (a metabolite of NO) at the doses 40 and 100 mg/kg may reflect the increased production of NO with nitrosative influence on the brain. 

Our results are in consistent with previous works in which the intoxication of ZnONPs are mediated through oxidative and nitrosative stresses [21,28,50,56]. The intracellular dissolution of ZnONPs and the liberation of ionic Zn^2+^ could also be responsible for its “toxicity” [57]. As mentioned before, ZnONPs have the ability to react with plasma and brain proteins [34]. ZnONPs have been established to be tightly bound mainly to ATP synthase in brain which lead to mitochondrial dysfunction and impaired ATP production. The relationship between mitochondrial dysfunction and ROS production is well documented and could be the main mechanism in causing nanotoxic effects in various cell lines [58]. Based on this information, the ZnONPs-induced oxidative stress with the subsequent toxic influences might be related to mitochondrial dysfunction by interacting with proteins in ATP production. 

NO and ROS are known to act cooperatively to induce the release of pro-inflammatory mediators [50,59,60], which in consistent with our results in which the oxidative and nitrosative effect following the 7 days exposure to 40 and 100 mg/kg of ZnONPs increased the brain concentrations of the pro-inflammatory cytokines; IL-1β and TNF-α. Following 7 days of exposure, the concentrations of IL-1β and TNF-α induced by the doses 40 and 100 mg/kg were significantly elevated compared to the corresponding concentrations after 24 h, with non-significant variations were detected at the dose 100 mg/kg/daily for 7 days compared to the dose 40 mg/kg/daily for 7 days. These results may suggest the inflammatory response-induced-“neurotoxicity of” ZnONPs may be time-dependent regardless of the administered dose. Similar results, although with higher dose (600 mg/kg), ZnONPs led to “neurotoxicity” following 7 days of oral exposure via oxidative and inflammatory mechanisms [50], therefore our results point to even lower doses could elicit “neurotoxicity” following oral ingestion. It has been proposed that the upregulated expression of the inflammatory mediators during oxidative and nitrosative stress response is triggered through the activation of the transcription factor nuclear factor kappa-B [21,61]. Interestingly, besides apolipoprotein E, the proteins involved in inflammation and complement activations were also detected in the surface of ZnONPs in brain homogenate [34] which may play a role in the inflammatory reaction after oral exposure. 

The present data revealed that exposure to 40 and 100 mg/kg/day of ZnONPs for 7 days causes DNA damage as evident from the data of DNA fragmentation and DNA comet assay. The increase in DNA tail length at both doses could refer to high percent of DNA strand break. This genotoxicity could be attributed to the oxidative and nitrosative effects induced by ZnONPs [21,56,62,63,64]. The inflammatory cytokines release could also contribute in DNA damage caused by ZnONPs [65]. Moreover, the lipid peroxidation’s products could react with DNA causing its damage [66]. Therefore, the highly pronounced genotoxicity observed with the dose 100 mg/kg/day for 7 days is possibly owing to the significant rise in lipid peroxidation at this dose compared to other three ZnONPs-treated groups.

DNA fragmentation is an important feature of cell apoptosis; therefore we, next, evaluated the apoptotic markers in ZnONPs exposed rats. Apoptosis is a process of programmed cell death that regulates cell renovation and elimination of injured cells [57]. However, cell death and impairment of tissues could be triggered by dysregulation of cell apoptosis, hence causing organ dysfunction [67]. Two major pathways are involved in cell apoptosis: The intrinsic (or mitochondrial) and extrinsic (or death receptor) signal transduction pathways, both of them end with the activation of caspase-3 which is the executioner apoptotic protein [67]. Extrinsic pathway starts by stimulation of death receptor, which is a member of tumor necrosis factor receptors superfamily, leading finally to the activation of caspase-3 [67]. In intrinsic pathway, the apoptotic stimuli lead to the opening of mitochondrial permeability transition pore with the liberation of cyt c, which finally results in activation of caspase-3. Caspase-3 is an inactive zymogen in the cytoplasm, which upon activation will trigger cell apoptosis via a signal transduction pathway. Fas is a death receptor that is constitutively expressed in a diversity of cell types, and mediates rapid apoptosis in response to certain stimuli, among them are the NPs; for example, nanosized TiO_2_ together with UVA radiation induces up-regulation of Fas and subsequent caspase activation and apoptotic cell death [68].

Although many in vitro studies demonstrated that treatment with ZnONPs led to reduced cell viability, increased apoptotic cells’ number and triggering of caspase-3 in different neural cells [29,69,70,71], whether the apoptotic pathway is enhanced in vivo following oral exposure remains controversial. Our in vivo study confirmed that oral exposure to ZnONPs (<100 nm, either at 40 or 100 mg/kg daily for 7 days) caused brain tissue apoptosis as indicated by augmented DNA fragmentation and the elevation of caspase-3, as well as increment of Fas was detected following the exposure to the high dose. Some underlying mechanisms may be implicated in ZnONPs-induced apoptosis. This includes the generation of ROS and the associated oxidative stress involving induction of lipid peroxidation in addition to the DNA damage [56,64,72]. Moreover, the exaggerated ROS production could lead to the opening of mitochondrial membrane permeability transition pore and, consequently the initiation of the apoptotic signaling pathway [73]. Similarly; several studies provide evidences for the role of ROS as potential inducers of mitochondrial dysfunction and subsequent apoptotic cell death [68,74]. Likewise, an in vitro investigation by Wang et al. [71] demonstrated that oxidative stress induced by ZnONPs, activates apoptosis through triggering JNK signaling pathway in cultured primary astrocytes. 

In addition, the elevated concentrations of TNF-α induced by ZnONPs after 7 days, suggest that ZnONPs may induce brain apoptosis via TNF-α mediated pathway. That could be clarified by, TNF-α has been reported to stimulate apoptosis through caspase activation pathways [75,76]. 

Heat shock proteins (HSPs) belong to a group of stress-responsive proteins which are extensively expressed in nearly all organisms [77]. Among different HSPs, HSP-70 is expressed at low levels under physiologic circumstances and has specific regulatory effects on cell growth, development, differentiation and cell death. HSP-70 is up-regulated following exposure to a variety of stresses and insults such as cellular energy depletion, oxidative stress and inflammation [78]. Consistent with this data, brain levels of HSP-70 were elevated after exposure to ZnONPs for 7 days as a consequence of the triggered oxidative stress and inflammation. In addition, it is demonstrated that HSP-70 is an inhibitor of apoptosis via inactivating caspase-3, and therefore could be cytoprotective [79,80,81]. The anti-apoptotic properties of HSP-70 have been attributed to its anti-oxidative action [82,83]. Accordingly, the high levels of HSP-70 in the current study following oral exposure of ZnONPs for 7 days could be a compensatory mechanism to counteract the ZnONPs-induced oxidative stress and apoptosis. 

Finally, the results of ZnONPs after 7 days are comparable to the toxicity induced by mercury which is a well-known neurotoxin. Depletion of GSH, increased lipid peroxidation, inflammation and oxidation of proteins and DNA in the brain are the main deleterious effects exerted by mercury in brain [84].

## 5. Conclusions

In conclusion, oral administration of low doses; 40 and 100 mg/kg/day of ZnONPs for 7 days have the ability to induce many deleterious effects in brain tissue, including oxidative stress, elevation of inflammatory cytokines, DNA fragmentation and apoptotic stimulation.

## Figures and Tables

**Table 1:** ZnONPs-treated groups and normal controls.

Groups	Dosage	Duration
Group I	40 mg/kg ZnONPs	24 h
Group II	100 mg/kg ZnONPs	24 h
Group III	40 mg/kg ZnONPs, daily	7 days
Group IV	100 mg/kg ZnONPs, daily	7 days
Control A	Normal Saline	24 h
Control B	Normal Saline, daily	7 days

**Table 2:** The comets DNA assay indices in normal control and ZnONPs-treated groups.

	Normal Control	G I (40 mg/kg/24 h)	G II (100 mg/kg/24 h)	G III (40 mg/kg/day for 7 days)	G IV (100 mg/kg/day for 7 days)
Tailed DNA %	4 ± 0.3	4.37 ± 0.29	5.03 ± 0.3	4.85 ± 0.28	5.6 ± 0.21^ a**b*^
Tail length (µm)	2.5 ± 0.17	2.7 ± 0.18	2.9 ± 0.146	3.3^ a*^ ± 0.157	4.18 ± 0.18 ^a***b***c*** d*^
Tail intensity (%)	2.12 ± 0.15	2.22 ± 0.28	2.19 ± 0.21	2.54 ± 0.216	3.38 ± 0.12 ^a**b**c**d*^
Tail moment (Unit)	4.66 ± 0.25	5.55 ± 0.293	5.07 ± 0.31	5.82^ a*^ ± 0.26	7.53 ± 0.263 ^a***b***c**d**^

Data are expressed as mean ± SEM. a: significantly different from normal control. b: Significantly different from G I. c: Significantly different from G II. d: Significantly different from G III. *: Statistically significant at *p* < 0.05. **: Statistically significant at *p* < 0.01. ***: Statistically significant at *p* < 0.001.
